# Population pharmacokinetic modelling of indium-based quantum dot nanoparticles: preclinical *in vivo* studies

**DOI:** 10.1016/j.ejps.2020.105639

**Published:** 2021-02-01

**Authors:** Elnaz Yaghini, Elisa Tacconi, Andrew Pilling, Paula Rahman, Joe Broughton, Imad Naasani, Mohammed R.S. Keshtgar, Alexander J. MacRobert, Oscar Della Pasqua

**Affiliations:** a UCL Division of Surgery and Interventional Science, University College London, Charles Bell House, 43-45 Foley Street, London, W1W 7TS, UK; b Clinical Pharmacology and Therapeutics Group, University College London, School of Pharmacy, BMA House, Tavistock Square, London, WC1H 9JP, UK; c ToxPath Consultancy Ltd, Church Road, Wingfield, Diss, IP21 5RA, UK; d Nanoco Technologies Ltd, 46 Grafton Street, Manchester M13 9NT, UK

**Keywords:** nanoparticles, quantum dots, pharmacokinetics, population pharmacokinetic modelling, biodistribution, toxicology

## Abstract

There is considerable interest in biomedical applications of quantum dot (QD) nanoparticles, in particular their use as imaging agents for diagnostic applications. In order to investigate the *in vivo* biodistribution and the potential toxicity of quantum dots (QDs), it is crucial to develop pharmacokinetic (PK) models as basis for prediction of QDs exposure profiles over time. Here, we investigated the *in vivo* biodistribution of novel indium-based QDs in mice for up to three months after intravenous administration and subsequently developed a translational population PK model to scale findings to humans. This evaluation was complemented by a comprehensive overview of the *in vivo* toxicology of QDs in rats. The QDs were primarily taken up by the liver and spleen and were excreted via hepatobiliary and urinary pathways. A non-linear mixed effects modelling approach was used to describe blood and organ disposition characteristics of QDs using a multi-compartment PK model. The observed blood and tissue exposure to QDs was characterised with an acceptable level of accuracy at short and long-term. Of note is the fast distribution of QDs from blood into liver and spleen in the first 24 h post-injection (half-life of 28 min) followed by a long elimination profile (half-life range: 47-90 days). This is the first study to assess the PK properties of QDs using a population pharmacokinetic approach to analyse *in vivo* preclinical data. No organ damage was observed following systemic administration of QDs at doses as high as 48 mg/kg at 24 h, 1 week and 5 weeks post-injection. In conjunction with the data arising from the toxicology experiments, PK parameter estimates provide insight into the potential PK properties of QDs in humans, which ultimately allow prediction of their disposition and enable optimisation of the design of first-in-human QDs studies.

## Graphical Abstract

**Figure:**
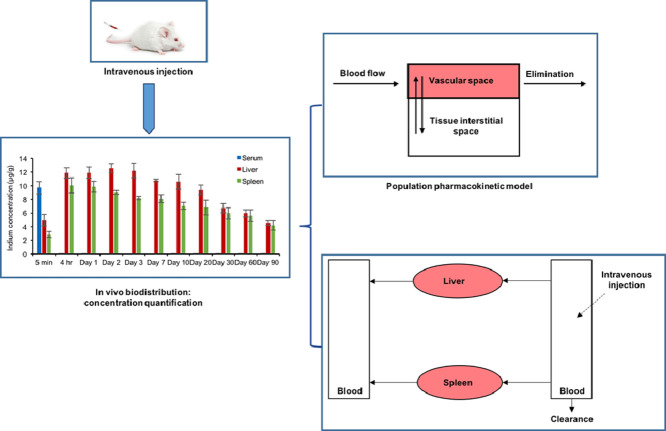

## 1 Introduction

Biomedical applications of quantum dot (QD) nanoparticles (NPs) such as medical diagnostics have attracted considerable interest over the past few years owing to their unique photophysical characteristics (Resch-Genger *et al.*, 2008; Yaghini *et al.*, 2009). However, their clinical application has been hindered by the use of restricted heavy metals such as cadmium in the QDs core, which has raised concerns about their toxicity (Lin *et al.*, 2011; Tchounwou *et al.*, 2012; Yaghini *et al.*, 2014; Lu *et al.*, 2016). Consequently, regulated heavy metal-free QDs have been developed, including indium-based QDs, to alleviate these concerns and render QDs fully biocompatible (Xu *et al.*, 2016). Recently, we have also demonstrated preclinical *in vivo* biocompatibility of indium-based QDs, which has prompted further interest in their clinical biomedical applications (Yaghini *et al.*, 2016; Yaghini *et al.*, 2018).

Among the many requirements for the progression of candidates into clinical drug development, understanding of *in vivo* pharmacokinetics is essential for translating preclinical data into relevant applications in humans (Valic & Zheng, 2019). In fact, the use of modelling techniques to characterise all relevant pharmacokinetic (PK) processes that determine systemic exposure and drug disposition (i.e., distribution and elimination) has been recognised as an important tool across different phases of drug development (Huntjens *et al.*, 2008; Bertrand & Leroux, 2012; Della Pasqua, 2013). Non-linear mixed effects modelling is a parametric modelling approach that attempts to extend the traditional modelling methods to explain variability arising from different subjects/sources (inter-subject variability) (Duffull *et al.*, 2011). Consequently, the estimation of parameters and corresponding distributions allows one to understand not only the overall characteristics of the population, but also quantify and identify individual differences in systemic exposure as well as in organ and tissue distribution due to inherent variation in physiological processes, such as haemodynamics and organ function (e.g. hepatic clearance). Model parameterisation is based on the assumption that pharmacokinetic processes can be described by mass transfer concepts and as such can be represented by a system of compartments (Meibohm & Derendorf, 1997; Ette & Williams, 2004).

The use of population pharmacokinetic modelling to analyse data from preclinical *in vivo* studies enables the assessment not only of the processes that determine the time course of QDs in blood, but also provides insight into tissue distribution and organ uptake, which in turn will be important for subsequent determination of diagnostic and therapeutic human doses, both in terms of safety, toxicity and theranostic efficacy of nanoparticles. In contrast to the use of empirical approaches for dose selection in humans based on safety factors, understanding of drug disposition and biodistribution along with the availability of PK parameter estimates provides the basis for a more robust prediction of the exposure in humans.

Despite the availability of various studies on tissue uptake kinetics and clearance, very few have focused on the characterisation of pharmacokinetics of QDs using a model-based approach (Lin *et al.*, 2008; Lee *et al.*, 2009; Arnold *et al.*, 2014; Liang *et al.*, 2016). Moreover, the few studies that have been carried out on QDs are restricted to cadmium-based products. From a methodological perspective, it is worth noting the use of physiologically-based PK (PBPK) models, which are often suited to describing mean exposure profiles, require considerably more experimental data to ensure accurate characterisation of the mass balance across relevant organs. Another important challenge for the use of PBPK models remains the limited knowledge of the role of potential active mechanisms associated with the transport and tissue distribution of nanoparticles (Lee *et al.*, 2009; Wang *et al.*, 2016).

The aim of the present study was to develop a multi-compartmental PK model to characterise the disposition profile of a novel water soluble red-emitting regulated heavy metal-free indium-based QDs in blood and major organs after intravenous administration of a clinically relevant dose to mice. Serum kinetics and tissue distribution including excretion kinetics of QDs into selected organs were investigated for up to 3 months. In addition, given the long elimination phase of QDs, which was observed in the *in vivo* biodistribution study, we have also conducted an in-depth toxicological evaluation of the same QDs in rats to investigate their biocompatibility and safety profile so as to consolidate with our previous toxicological studies on related cadmium-free QDs as basis for further progression of the moiety to the clinic. A model parameterisation was identified that allows the evaluation of short and long term of QDs disposition characteristics. As far as we are aware, no previous study has used a model-based approach to describe the pharmacokinetics of QDs in a preclinical species.

## 2 Methods

### 2.1 Synthesis and characterisation of quantum dot nanoparticles

The indium-based QDs (PEGylated bio CFQD^Ⓡ^ nanoparticles) were synthesized and functionalised using the same processes described in our previous work (Yaghini *et al.*, 2016), except for the inclusion of polyethylene glycol, which has a longer chain (PEG5000) during the surface treatment step to enhance the physicochemical properties and conjugation liability. The resulting nanoparticles have surface carboxyl groups available for conjugation, which confer a negative surface charge and has a mixture of PEG 5000 and PEG 2000 chains. The peak photoluminescence emission wavelength was 635 ± 5 nm, as measured using a fibre optic CCD spectrometer (USB4000, Ocean Optics Inc.).  The hydrodynamic size was 15.5 ± 1 nm with a polydispersity index (PDI) <0.29, as measured using dynamic light scattering (Malvern Zetasizer µV system) in HEPES 20 mM buffer at pH 6.0. The increased hydrodynamic size in comparison with the previously studied bio CFQD^Ⓡ^ particles (12.1 nm) is mainly due to the longer emission wavelength and the inclusion of larger PEG chains.

### 2.2 Animal experiments

Female Balb/c mice and Wistar rats were purchased from Charles River. All procedures were conducted with Home Office licence approval.

### 2.3 *In vivo* biodistribution, blood clearance and excretion studies

Female Balb/c mice were used for the *in vivo* biodistribution and pharmacokinetic studies of QDs. In order to ensure accurate characterisation of the disposition properties, QDs were administered intravenously. Ten groups of animals (n = 3) were used: nine groups were administered intravenously via the tail vein with 200 µL of the QDs in PBS at a dose of 20 mg/kg. Another group of animal served as the control group and were injected intravenously with 200 µL of PBS. Mice were sacrificed at 5 min, 1 h, 4 h, 1, 3, 10, 30, 60 and 90 days after the injection and blood and various organs including brain, thymus, lung, heart, liver, spleen, kidney, intestine, muscle and skin were collected to determine the distribution of QDs and indium concentration into tissues and organs. For the measurement of the clearance and half-life of QDs in serum, another set of Balb/c mice (n = 3) were injected intravenously with QDs at the same dose and the samples were collected at 5, 20, 40, 60 and 120 min post-injection. The bioanalysis section (section 2.5) describes the experimental details for quantifying the indium concentration in serum.

The *in vivo* excretion of QDs was characterised after a dose of 20 mg/kg using a set of 8 female Balb/c mice. Another three mice were used as control group and received PBS only. The urine and faeces samples (in triplicate) were collected at 1, 3, 7, 10, 20, 30, 60 and 90 days after QDs injection.

### 2.4 *In vivo* toxicology study

Female Wistar rats were used for the *in vivo* toxicology study. Rat models offer advantages over mice for such studies, in particular larger blood volumes and organ sizes that facilitate multiple histological and biochemical analyses. Nine groups of animals (n = 3) were included in the *in vivo* toxicology study: six groups were injected intravenously with 500 µL QDs solution in PBS at a concentration of 12 mg/kg and 48 mg/kg, whereas three animals were used as control, receiving 500 µL PBS solution intravenously. The rats were sacrificed at 24 h, 1 and 5 weeks after the injection. Major tissues from each animal, including brain, lung, heart, liver, spleen, kidney and skin were collected, fixed in 4% formalin, sectioned and stained (haematoxylin & eosin). Histopathology assessments of the tissues were performed by a veterinary pathologist; blood or tissue concentrations of QDs were not measured. Blood samples were collected for the full haematological analysis (details described in bioanalysis section 2.5), including white blood cells (WBC), red blood cells (RBC), haemoglobin (HGB), haematocrit (HCT), mean corpuscular volume (MCV), mean corpuscular haemoglobin (MCH), mean corpuscular hemoglobin concentration (MCHC) and red blood cell distribution width (RDW). In addition, a biochemical analysis was performed to assess liver and kidney function, including blood urea nitrogen (BUN), creatinine (Crea), aspartate transaminase (AST), alanine transaminase (ALT), alkaline phosphatase (ALP) and total protein (TP).

### 2.5 Bioanalysis

#### 2.5.1 Quantifying the indium concentration in serum samples

Following collection of blood samples at various post-injection times (as described in section 2.3), the samples were transferred into Eppendorf tubes and left at room temperature for 10 min. Subsequently, the serum was separated by centrifugation of blood for 10 min at 14000 rpm. Serum aliquots were then transferred into a cryovial and stored at -80°C prior to inductively coupled plasma mass spectroscopy (ICP-MS) measurements for quantification of the indium content.

#### 2.5.2 Biochemical and haematological analysis

Blood samples were collected from each rat at various post-injection times following intravenous injection of QDs. Half of each sample was immediately transferred into the tubes containing anticoagulants and placed on a blood roller mixer. The other half of the remaining blood samples was transferred into Eppendorf tubes and left at room temperature for 10 min. Afterwards, the tubes were centrifuged for 10 min at 14,000 rpm to separate the serum for biochemical analysis, which was performed on the same day at the Pathology and Diagnostic Laboratories of the Royal Veterinary College, London.

### 2.6 Quantification of QDs uptake in organs

Samples of 0.1 g of each tissue from mice in triplicate were prepared and digested by the addition of 1 mL 70% nitric acid (HNO_3_) as detailed previously (Al-Jamal *et al.*, 2009; Yaghini *et al.*, 2016). ICP-MS was employed to quantify the amount of indium (In) in serum samples and in each organ. This standard technique for quantification of inorganic nanoparticles in tissue was selected because of its high sensitivity (Al-Jamal *et al.*, 2009). For all measurements, nitric acid blank, blank tissue samples, spiked samples with known QDs for calibration and indium standards were prepared and tested concurrently with test samples. The tissues from the control mice without QDs injection were digested in a similar manner.

### 2.7 Statistical analysis

To assess the statistical significance of potential toxicological findings, a two-sample *t* test for unknown and unequal variances was used, comparing each QD-injected group to the related control group at the same dose. The error bars shown are the standard deviations (SD). Results were considered significant for *P* < 0.05.

### 2.8 Pharmacokinetic modelling

#### 2.8.1 Evaluation of QDs disposition characteristic in blood

The pharmacokinetic analysis was carried out in NONMEM version 7.3 (Icon Development Solutions, USA). Parameter estimation was based on the first order conditional estimation method with interaction (FOCE-I) (Hooker *et al.*, 2007). Population PK model building was performed in a stepwise manner using serum indium concentration data. First, a base model (no covariates) was built using one, two or more compartments. This was accomplished by initially identifying the appropriate structural model, regardless of error. Subsequently, suitable stochastic models of between-subject variability (BSV) and residual variability (RV) were evaluated. A parameter value of a subject *i* (*Θ_i_* = post hoc value) is given by the following equation:

(1) $$\Theta i=\Theta_{TV}*e^{\eta i}$$

where *θ_TV_* is the typical value of the parameter in the population and *ηi* is assumed to be a random variable with zero mean and variance *ω^2^*.

Residual variability was initially described with a proportional error model. Other residual error models, e.g., additive or additive plus proportional, were considered based on the evidence of bias or trends in model diagnostics and goodness-of-fit plots. This means that for the *j^th^* observed concentration of the *i^th^* individual, the relation is:

(2) $$Y_{ij}=P_{ij}*\left(1+\varepsilon_{ij}\right)$$

where *P_ij_* is the predicted concentration and *ε_ij_* a random variable with mean zero and variance *σ^2^*.

Model selection was based on statistical criteria, as assessed by changes in the objective function value (OFV). Changes in the objective function after the addition of a parameter approximate a $X^{2}\;$ distribution with one degree of freedom. Hence, a parameter is considered statistically relevant (*P* < 0.05) and included in the model if OFV decreased by > 3.84 (Hooker *et al.*, 2007). Model building criteria also included successful minimization, standard error of estimates, number of significant digits, termination of the covariance step and correlation between model parameters. Goodness-of-fit was assessed by graphical methods, including population predicted vs. observed concentrations, and conditional weighted residuals vs. observed concentrations and time. R version 3.6.1 and R-Studio user interface were used for all data manipulation and subsequently for creating graphical and statistical summaries.

**Fig. 1:**
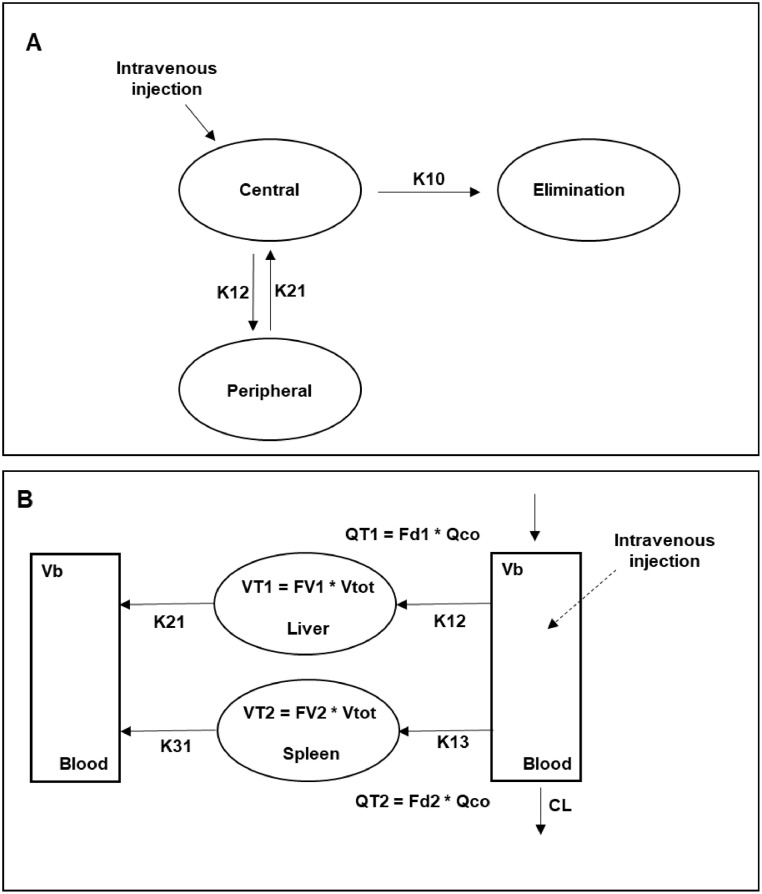
(A) Diagram describing the structural pharmacokinetic model for quantum dots (QDs) disposition in blood. The two-compartment model indicates that distribution of QDs from organ and structures that are highly vascularized (i.e., central compartment) is not instantaneous; the peripheral compartment represents tissues and organs in which the QDs distribution is limited by haemodynamic and/or permeability factors. (B) Structural model describing QDs organ disposition. Each compartment represents a single organ or tissue, namely blood, liver and spleen (see Table 2 for parameter definitions). Vb: blood volume, Vtot:total body volume, VT1: volume of distribution in the liver, VT2: volume of distribution in the spleen. VT1 = FV1*(Vtot – Vb); VT2 = FV2*(Vtot – Vb).

The final model describing the pharmacokinetics of QDs in serum was a two-compartment model with first-order elimination. Given the sparse sampling scheme and limited number of animals per group, it was not possible to identify between-subject variability for model parameters. Whilst model compartments do not correlate strictly with anatomical structures, it can be assumed that the central compartment represents the space within the circulatory system that is highly vascularized, where the distribution of the QDs is instantaneous upon intravenous injection. By contrast, the peripheral compartment usually represents organs or structures in which distribution is slow relative to the central compartment, usually due to haemodynamic or permeability factors (Fig. 1A).

#### 2.8.2 Evaluation of QDs disposition characteristics in relevant organs

The second objective of this study was to evaluate the QDs distribution into tissues and their disposition over a period of three months. A compartmental PK model was used to estimate the equilibration kinetics of QDs between organs (blood, spleen, liver) at various post-injection times taking into account the initial distribution phase immediately after administration. QDs equilibration kinetics was investigated in spleen and liver since the majority of the injected QDs accumulated in these two organs.

To describe organ disposition, a three-compartment model was used that accounted for the distribution of QDs from blood into liver and spleen and subsequent re-equilibration processes (Fig. 1B). Given the sparse sampling scheme (destructive sampling), a single compartment was assigned to each organ/tissue (i.e. blood, spleen and liver compartments) (Cao & Jusko, 2012). The first order conditional estimation (FOCE) method was implemented in NONMEM using the ADVAN 6 subroutine. The model structure is explained in Fig. 1B and is described according to the following differential equations:

(3) $$\frac{dC1}{dt}\;\left(blood\right)=k21*C2+k31*C3-k12*C1-k13*C1-ke*C1$$

(4) $$\frac{dC2}{dt}\;\left(liver\right)=k12*C1-k21*C2$$

(5) $$\frac{dC3}{dt}\;\left(spleen\right)=k13*C1-k31*C3$$

where C1 is the concentration of QDs in the blood compartment, C2 and C3 are QDs concentrations in tissue compartments 1 *(VT1)* and 2 *(VT2), ke* is first order elimination rate constant from the blood compartment. k12, k21, k13, k31 are intercompartmental rate constants. As bioanalysis was performed in serum, it was assumed that 1) equilibration kinetics between serum and blood is instantaneous and 2) QDs uptake by erythrocytes is negligible.

The key feature of this model are the intercompartmental rate constants because they explain the biodistribution of QDs between the organs. Each intercompartmental rate was calculated using the following equations:

(6) $$k12=\frac{QT1}{Vb}$$

(7) $$k21=\frac{QT1}{V1}$$

(8) $$k13=\frac{QT2}{Vb}$$

(9) $$k31=\frac{QT2}{VT2}$$

(10) $$\;ke=\frac{CL}{Vb}$$

(11) QT1=Fd1*QCO

(12) QT2=Fd2*QCO

Where *QCO (Q-Cardiac Output)* is the blood flow and *Fd1* and *Fd2* are fractions of *QCO* for *VT1* and *VT2*, respectively*. VT1* and *VT2* describe the volume for liver and spleen compartments and are calculated trough the total volume (*Vtot*) and the corresponding fractions (*FV1, FV2*).

### 2.9 Pharmacokinetic model validation

The development and validation of the final pharmacokinetic models were based on graphical and statistical methods. The goodness-of-fit (GOF) plots are graphical summaries that describe how well a model fits a set of observations. It also provides insight into the discrepancy between observed values and model predictions. GOF plots included conditional weighted residuals (CWRES) vs. population predictions and conditionals weighted residuals (CWRES) vs. time. CWRES vs. population predictions and CWRES vs. *time* represent a way to assess the presence of bias or trends, which would indicate model misspecification. CWRES are defined as:

(13) $$CWRES=\frac{Yi-E_{FOCE}\;\left(Yi\right)\;}{\sqrt{COV_{FOCE}\;\left(Yi\right)}}$$

where $E_{FOCE}\;\left(Yi\right)\;$ and $COV_{FOCE}\;\left(Yi\right)$ represent the expectation and covariance matrices of the model linearized using the FOCE method. Under the statistical assumptions made for the modelling approach, the CWRES should follow a normal distribution N ∼ (0,1) and be independent (Hooker *et al.*, 2007).

Despite the limited sample size, bootstrapping was used as an attempt to obtain confidence intervals for model parameter estimates and evaluate model stability. The bootstrap procedures were performed in PsN v3.5.3 (University of Uppsala, Sweden) (Lindbom *et al.*, 2004), which automatically generates a series of new data sets by sampling individuals with replacement from the original data pool, and fitting the model to each new data set.

## 3 Results and Discussion

### 3.1 Characterisation of QDs

**Fig. 2:**
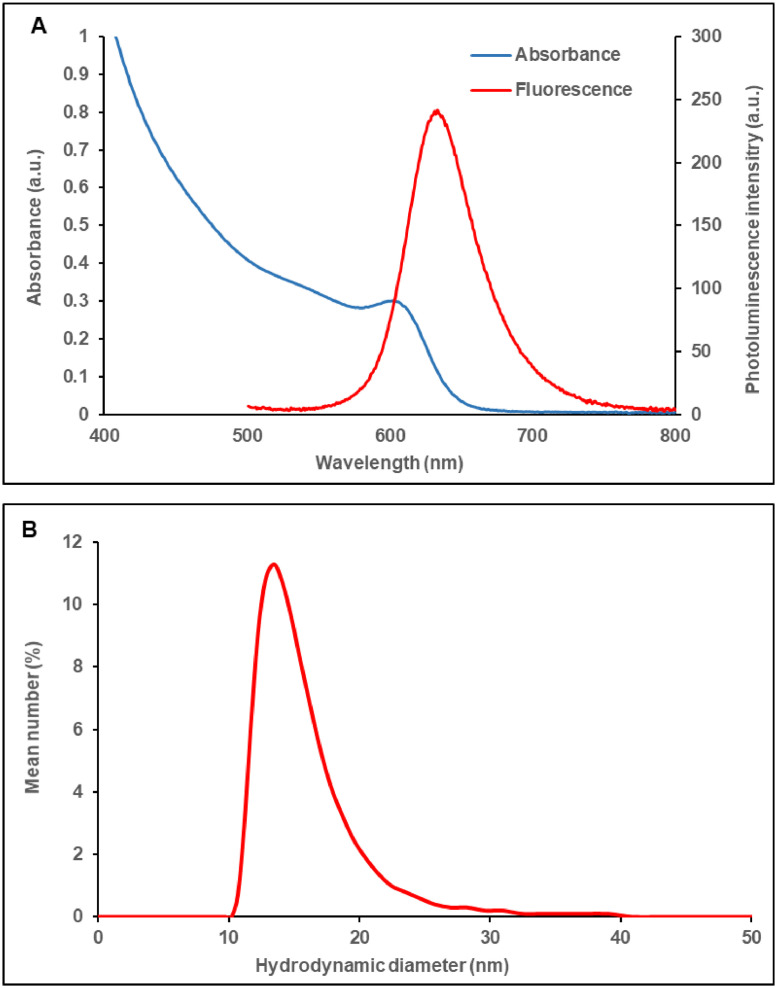
Absorbance and photoluminescence spectra (A); hydrodynamic size using dynamic light scattering (DLS) (B) of the PEGylated QDs. Measurements were performed on aqueous solutions of the quantum dots.

As shown in Fig. 2A, the photoluminescence peak of the QDs used in this study is 20 nm longer than that of the previously studied (Yaghini *et al.*, 2016) QDs (635 nm vs. 615 nm, respectively). This means that the PEGylated QDs of this study have a lower bandgap and a larger particle dimension. Indeed, the hydrodynamic size measured by dynamic light scattering (Fig. 2B) is ∼ 3 nm larger than the previous particles (15.1 nm vs. 12.2 nm, respectively).

### 3.2 *In vivo* biodistribution studies

Following systemic administration, nanoparticles are commonly cleared from the body through renal, hepatobiliary, and mononuclear phagocyte system (MPS) (Bertrand & Leroux, 2012; Ehlerding *et al.*, 2016; Zhang *et al.*, 2016). It is known that if nanoparticles (and their degradation products) are less than 6 nm in size, they can be cleared from the body through renal clearance within hours to days after administration (Choi *et al.*, 2007). Larger diameter non-degradable nanoparticles are more likely to be taken up and retained by MPS cells, including Kupffer cells, liver sinusoid endothelial cells (LSECs), splenic red pulp and marginal zone macrophages. Apart from MPS sequestration, other factors such as the tissue blood supply and the vascular permeability are also involved in the amount of nanoparticles that accumulate in tissues. The processing of nanoparticles in the MPS depends on their composition. In general, organic nanoparticles such as polymeric and liposomes are readily degraded in the MPS and if their degradation products are smaller than the renal threshold (less than 6 nm) then they can be eliminated through the renal pathway via the urine (Choi *et al.*, 2007). For larger nanoparticles, hepatobiliary or MPS clearance is more likely. However, inorganic nanoparticles such as gold, QDs and iron oxide nanoparticles have relatively more stable cores compared to organic nanoparticles and have been shown to become sequestered in the MPS organs for extended periods. Once nanoparticles are processed inside the MPS cells, the intact nanoparticles or their degradation products can be excreted in bile.

**Fig. 3:**
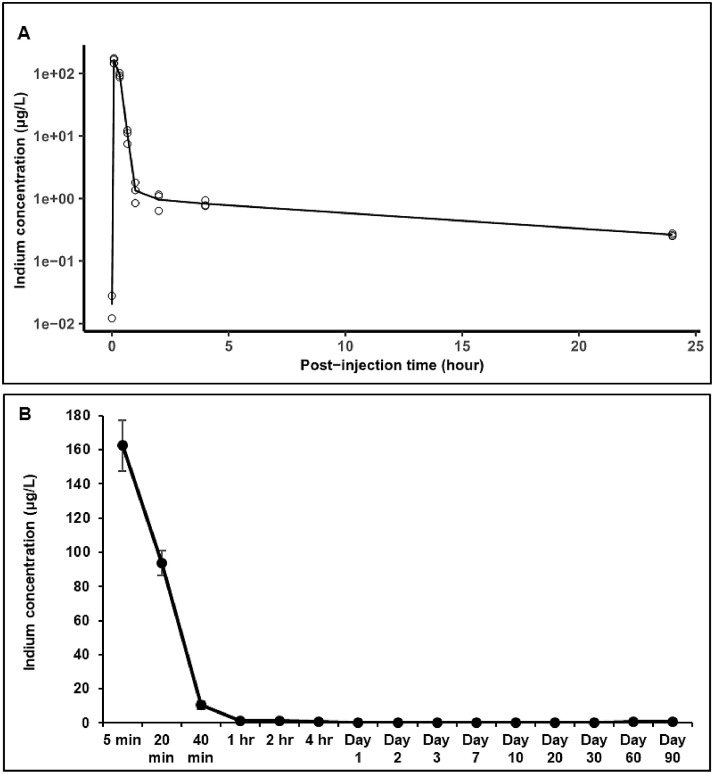
In vivo quantum dots (QDs) blood disposition (A) over a period of 24 hours and over a period of 90 days (B), in Balb/c mice following intravenous administration of the QDs at a dose of 20 mg/kg. Indium concentrations in the blood were measured at different time points after injection using ICP-MS (n = 3). Open circles show the observed indium concentrations and the black line corresponds to the averaged experimental fit to the data points. The rapid decrease at short times highlights the initial rapid distribution (from blood into tissues), which is followed by a much slower elimination phase.

In this study, the analysis of indium levels showed that the majority of QDs were present in blood during the first few mintues after intravenous administration (Fig. 3A). After 5 minutes post-injection, the indium levels in serum decreased rapidly from 162.48 µg/L to 93.93 µg/L and 10.36 µg/L at 20 minutes and 40 minutes post-injection, respectively (Fig. 3B). By 24 hr post-injection only negligible amounts of indium remained in blood (0.24 µg/L). As shown in Fig. 3A, the indium levels in blood decreased further whereby 3 days post-injection only trace amounts of indium were detectable (0.095 µg/L). Afterwards, indium concentrations in blood fluctuated over time, reaching levels of 0.78 µg/L after 90 days post-injection. This slight increase in the indium levels was due to equilibration/redistribution of QDs between tissues and the vascular system over the period of three months post-injection. These finding are similar to our recent *in vivo* study with bio CFQD^Ⓡ^ nanoparticles on Lister Hooded rats, where following systemic administration of QDs, the majority of QDs accumulated into the liver and spleen and were excreted from the body gradually over a period of three months (Yaghini *et al.*, 2018). In that study, the QDs were slightly smaller with a mean hydrodynamic diameter of 12.2 nm and for PEGylation only PEG 2000 chains were employed.

**Fig. 4:**
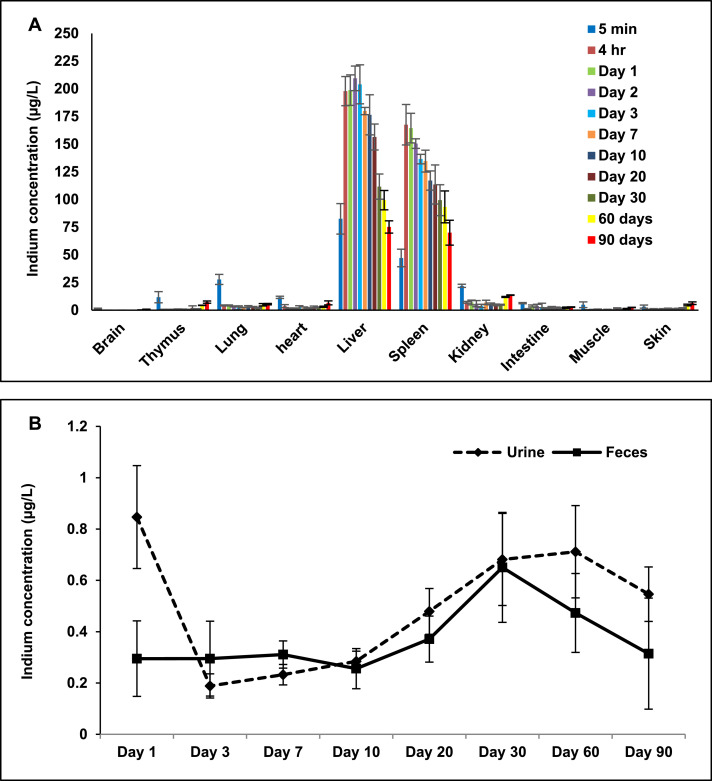
*In vivo* biodistribution (A), and excretion (B) analysis over a period of 90 days in Balb/c mice following intravenous administration of the QDs at 20 mg/kg. The indium concentration in the organs, faeces and urine samples was measured at different time points after injection using ICP-MS (n = 3).

The serum concentration vs. time-curve suggests fast distribution within organs which are highly vascularized, where indium can be detected already at 5 minutes after injection. Subsequently, the QDs accumulated primarily into MPS cells and in the liver and spleen (Fig. 4A). Indium concentrations in the spleen and liver increased gradually over time and reached a peak at 4 hours (167.46 µg/L) and day 2 (209.39 µg/L) respectively. Afterwards, indium levels decreased over time in the liver and spleen, where, respectively, 75.13 µg/L and 69.98 µg/L indium were still present on day 90.

Trace quantities of indium were also detected in the intestine suggesting that some QDs were excreted via hepatobiliary route. Evidence for hepatobiliary excretion was further supported by elemental analysis of faeces. As shown in Fig. 4B, small amounts of indium were detected in the faeces at various post-injection times. The mean hydrodynamic diameter of the QDs used in this study was 15.5 nm which is above the threshold for direct renal clearance. Consequently, only small quantities of indium were detected in the kidney, with levels slightly increased at 60 and 90 days (11.89 µg/L and 13.6 µg/L, respectively) (Fig. 4A). This suggests that some QDs underwent intracellular degradation and released degraded precursors containing indium for renal excretion. Further evidence for the urinary excretion of QDs was provided by elemental analysis of urine samples where trace amounts of indium were detected at various post-injection times (Fig. 4B). Only a small amount of indium was detected in the remaining tissues at different post-injection times. Our previous biodistribution study did not include excretion measurements.

### 3.3 Population pharmacokinetic model

The population pharmacokinetic model was developed to investigate the kinetics of QDs based on the experimentally measured concentration in blood, and two organs exhibiting high uptake, namely liver and spleen. We adopted essentially a hierarchical modelling approach where the process is represented by a system of compartments, in which drug equilibrates according to mass balance principles. The advantage of the compartmental approach is the ability to describe in more details how the concentration changes with time and to predict it at any given time and in each compartment. Based on the observed blood concentration profiles, two models were developed and refined to describe the pharmacokinetics of the QDs and evaluate their disposition characteristics: (a) in blood up to 24 hours after QDs injection, and (b) other relevant organs up to 3 months after QDs injection.

**Fig. 5:**
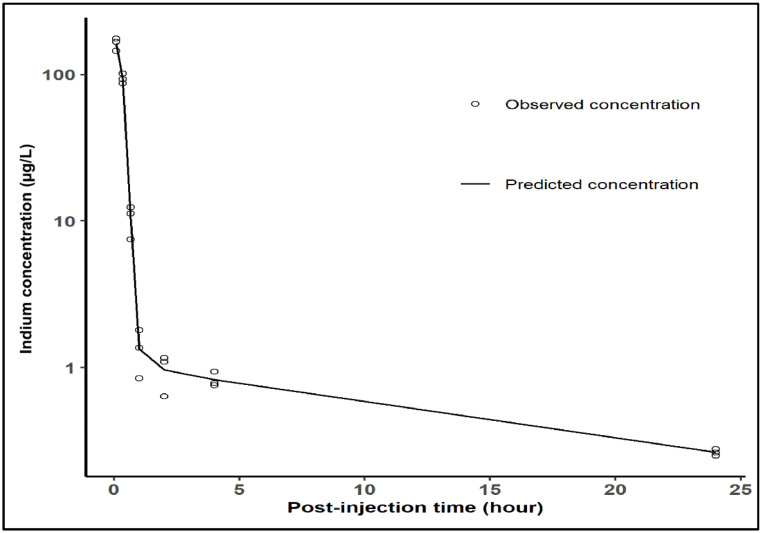
Population predicted pharmacokinetic profile in blood over time (up to 24 hours). The experimental data are shown as open circles, whereas the black line represents population predicted concentrations of quantum dots (QDs).

The initial disposition of QDs in blood was best described by a two-compartment model, in which rapid decrease in indium concentration takes place, indicating quasi-instantaneous transfer from the central (circulation) into the peripheral compartment (body tissues). A second, much slower re-equilibration and elimination process is observed, since the agent must first diffuse back from the tissues to blood. Residual variability was characterised by a proportional and additive error model. The terminal half-life in blood was calculated to be 28 minutes (Fig. 5), similar to the value of 29 minutes reported in Gao et al. study using PEGylated InAs/InP/ZnSe QDs in mice (Gao *et al.*, 2010). Table 1 summarises the pharmacokinetic results in blood based on data recorded up to 24 hours. Coefficient of variation (% CV) values indicate acceptable precision of the estimates.

**Table 1:** Population pharmacokinetic parameters describing QDs disposition characteristics in blood (CL: clearance; V1: volume of distribution in central compartment; V2: volume of distribution in peripheral compartment; Q: the inter-compartmental clearance, CV: coefficient of variation, T_1/2_: elimination half-life).

Parameters	Final model estimate	% CV
*CL (L/h)*	0.0416	12
*V1 (L)*	0.0027	9
*Q (L/h)*	0.498	21
*V2 (L)*	0.068	17
*Proportional error*	0.0686	8
*Additive error (ng/L)*	0.05 (38%)	12
*T_1/2_ (min)*	28	-

**Fig. 6:**
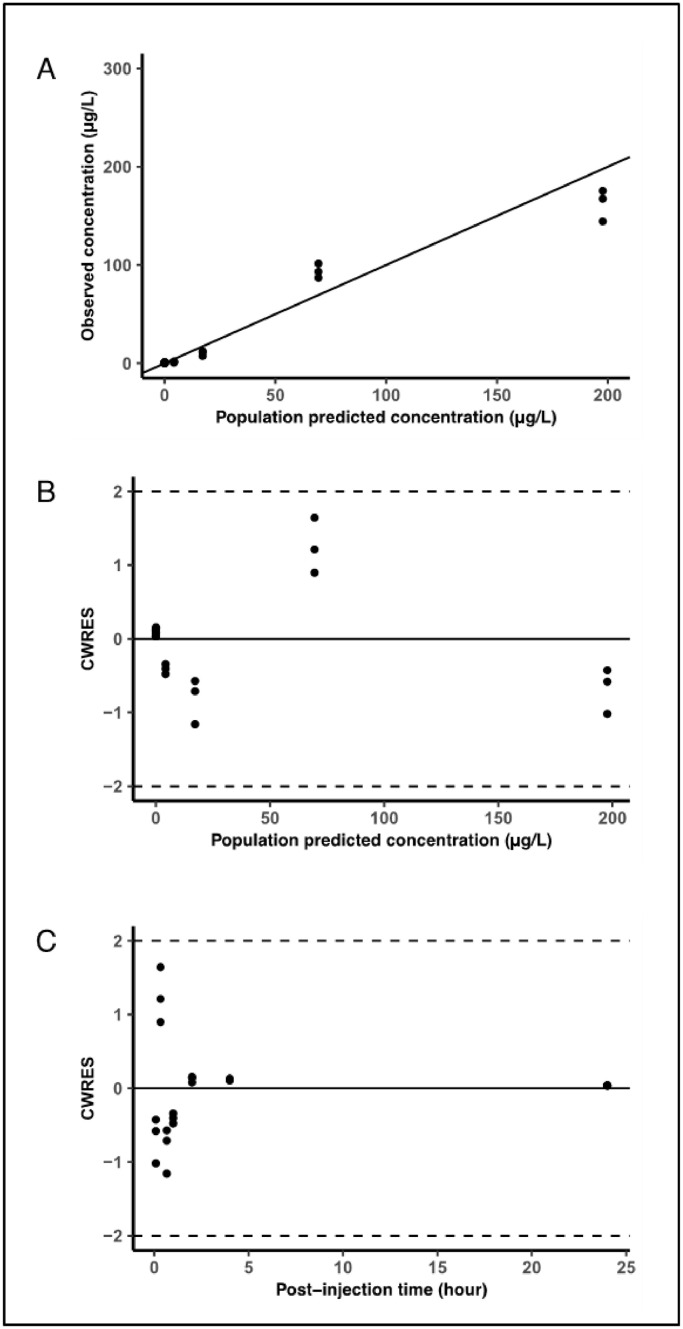
Goodness-of-fit plots. (A): shows observed vs population predicted concentration (µg/g), (B): Conditional weighted residuals (CWRES) vs population predicted concentration (µg/g) and (C): CWRES vs time (hours).

Overall model performance was deemed satisfactory. The population predicted profile and goodness-of-fit plots revealed that the model provided an adequate and un-biased description of the experimental data as shown in Fig. 6.

In addition to the short half life in blood, volume of distribution (V1 and V2) estimates indicate that QDs distribute well beyond total body water, reflecting tissue uptake. This implies that despite the high clearance values (i.e. higher than liver blood flow), QDs are not eliminated from the body, but promptly removed from the blood compartment.

To describe QD distribution into organs (liver, spleen), data from samples collected over a period 3 months were fitted with three-compartment model. Given the use of destructive samples, interindividual variability could not be estimated for model parameters describing the disposition into organs and tissues.

**Fig 7:**
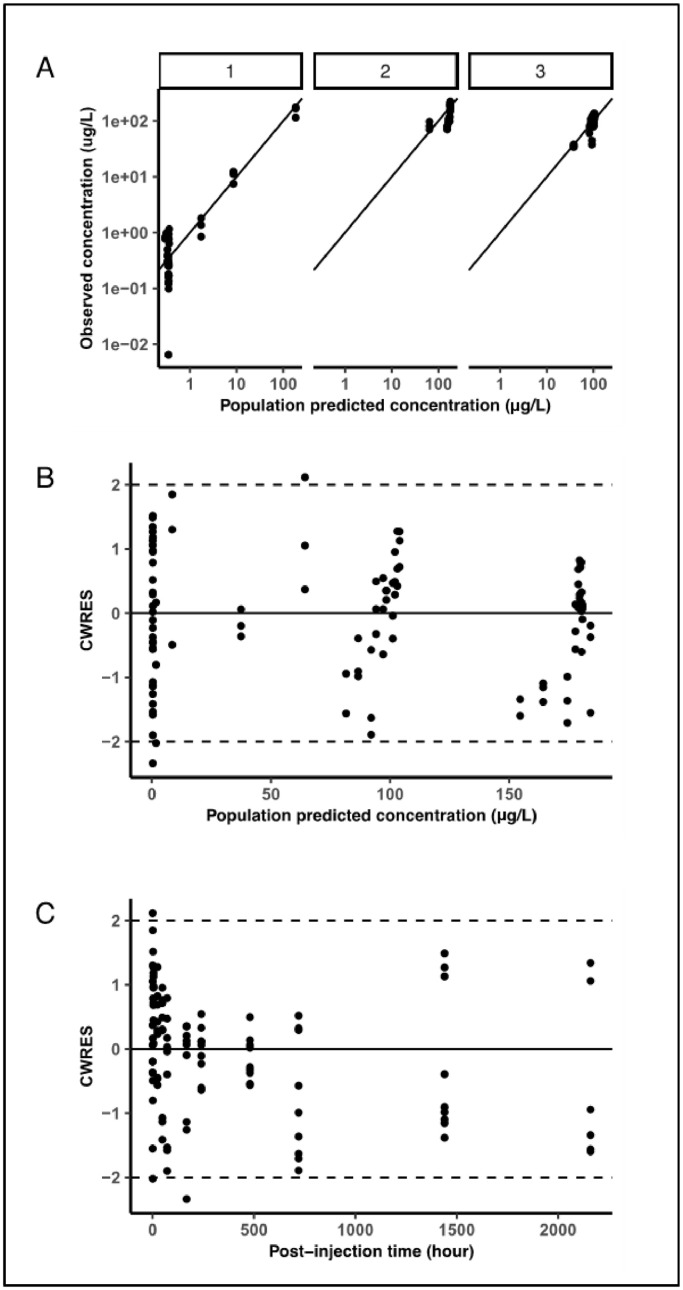
Goodness-of-fit plots. Panel A shows observed vs population predicted concentration (µg/L) in three different compartments (1 = Blood, 2 = Liver, 3 = Spleen); Panel B: Conditional weighted residuals (CWRES) vs population predicted concentration and Panel C: CWRES vs time (hours).

**Fig 8:**
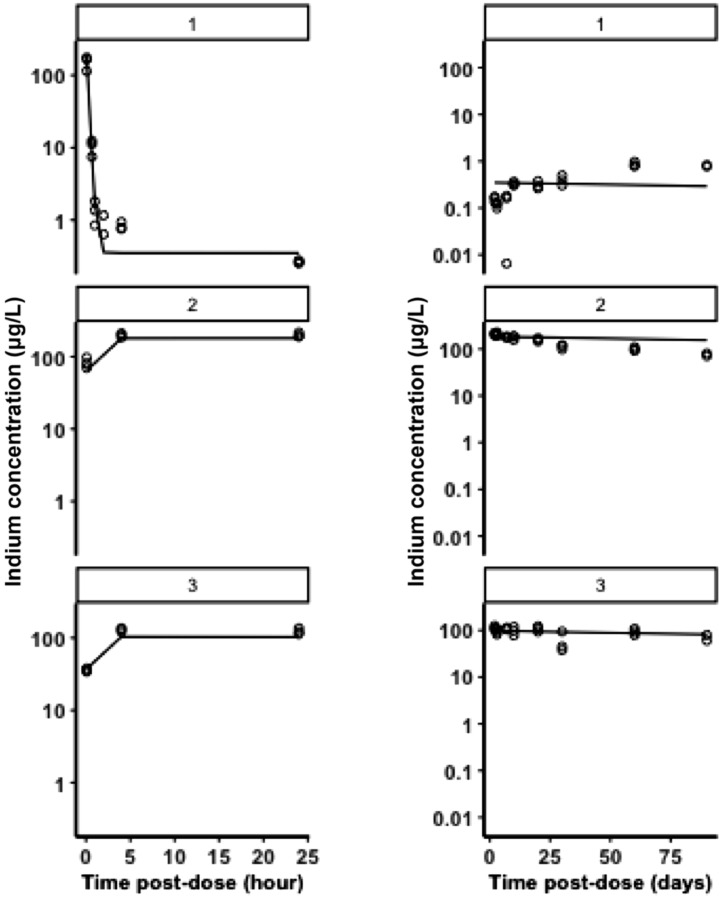
Population predicted pharmacokinetic profiles and observed concentrations (µg/L) in three different compartments (1 = Blood, 2 = Liver, 3 = Spleen); Left panels show tissue kinetics equilibration during the first 24 hours after administration, whilst right panels describe tissue (re-)distribution and elimination from blood over the period of 90 days post-dose. The experimental data are shown as open circles, whereas the black line represents population predicted concentrations of quantum dots (QDs) in each compartment.

Residual variability was characterised by a proportional and additive error model. The elimination half-life in blood derived from this model was 47 days, indicating very slow re-distribution and re-equilibration. Similarly to the estimates obtained for the initial 24 hours, coefficient of variation (% CV) values indicate acceptable precision of the estimates. An overview of the pharmacokinetic parameter estimates is presented in Table 2. Model diagnostics revealed acceptable goodness-of-fit for the final model. As shown in Fig. 7, the population predictions for blood and each organ (1 = Blood, 2 = Liver, 3 = Spleen) were unbiased. Fig. 8 represents the population predicted pharmacokinetic profiles and observed concentrations (µg/L) in three different compartments (1 = Blood, 2 = Liver, 3 = Spleen).

**Table 2:** Population pharmacokinetic parameters describing QDs disposition characteristics in mice based on a three-compartment model. Intercompartmental rates represent the re-distribution of QDs between blood, liver and spleen. CL = clearance; Vb = volume of distribution in blood; ke = elimination rate constant from blood; T_1/2_ = elimination half-life from blood or tissue; QT1, QT2 = intercompartmental clearance; VT1, VT2 = volume of distribution in tissue (spleen and liver); QCO = cardiac output; Vtot = total volume; K_21_ = equilibration rate constant from spleen to blood; K_12_ = equilibration rate constant from blood to spleen; K_31_ = equilibration rate constant from liver to blood; K_13_ = equilibration rate constant from blood to liver.

Parameters	Tissue	Final Model Estiumate	% CV
CL (L/h)	Blood	0.0021	29
Vb (L)	Blood	3.41	33
ke (1/h)	Blood	0.0006	-
T_1/2_ (day)	Blood	47.0	-
QT1 (L/h)	Liver	0.0018	35
VT1 (L)	Liver	0.13	34
T_1/2_ (day)	Liver	2.0	-
QT2 (L/h)	Spleen	0.1606	23
VT2 (L)	Spleen	0.22	34
T_1/2_ (day)	Spleen	1.0	-
QCO (L/h)	Blood flow	1.07	42
Vtot (L)	Total volume	3.65	34
K21 (1/h)		0.014	23
K12 (1/h)		0.0005	27
K31 (1/h)		0.73	32
K13 (1/h)		0.047	34

Table 2 also shows the estimated values for the intercompartment rate constants (Fig. 1B), which describe the re-distribution of QDs between blood, liver and spleen. These rate constants were used to calculate the respective elimination half-lives from the organs*.* Whilst a full physiological interpretation of the parameters may not be possible, equilibration rate constants show that indium concentrations re-equilibrate slowly, with elimination half-life from blood reaching 47 days. This contrasts with the values observed for liver and spleen (2 days and 1 day, respectively), which indicates indium continues to re-equilibrate with other tissues, rather than undergoing excretion immediately after reaching blood.

In summary, in this study we have demonstrated the use of a nonlinear mixed effects modelling approach to describe the *in vivo* biodistribution of indium-based cadmium-free quantum dot nanoparticles in mice. Given the differences in equilibration kinetics in different organs/tissues, a two-step approach was required to evaluate the disposition characteristics of QDs: firstly, to describe the immediate distribution and elimination from blood during the first 24 hours after injection; secondly, to characterise tissue redistribution and slow re-equilibration kinetics over the period of three months. In the first step, we have parameterized only the processes associated with disposition in blood using a two compartment model, which included estimates of clearance and elimination half-life. Due to the sparseness of samples and major difference in time scale, a distinct, separate model was required to describe tissue disposition and QDs re-equilibration kinetics in spleen and liver.

Of interest are the intercompartmental rate constants, which provide insight into organ-specific differences in biodistribution. We have observed differences in the half-life estimates in blood after 24 h and 3 months post-injection (i.e., 28 min vs. 47 days). This is explained by the fact that half-life estimates obtained from data collected within the first 24 hours reflect distribution processes that determine QDs uptake into organs and tissues. Clearly, elimination half-life estimates obtained from data collected over the period of 3 months suggest that indium excretion from the body is significantly slower, and dependent on re-equilibration between organs, tissues and blood. The estimates of 47 days need therefore to be considered with caution, as these values imply a washout time of approximately 6 months. Unfortunately, experimental data were not available beyond 90 days post-injection. Most likely, this long blood half-life results from the slow release of indium into the circulation following degradation of the QDs inside phagocytic cells of the reticuloendothelial system.

## 4 *In vivo* toxicology

The results from the biodistribution and PK studies prompted us to conduct an in depth toxicology study of the PEGylated QDs nanoparticles following their systemic administration. Standard haematological and biochemical tests together with histological analysis of various tissues were employed for the toxicology assessments. Rats were injected intravenously with QDs at 12 or 48 mg/kg (n = 3) and sacrificed at 24 h, 1 week and 5 weeks after QDs injection for blood, serum and organ collection. Another set of rats were used as the control group and were injected with PBS intravenously. In our previous studies using CFQD^Ⓡ^ nanoparticles, which had a similar core/shell compostion but bore a modified PEG coating, no adverse effects were observed following intravenous injection of the QDs (Yaghini *et al.*, 2018). Likewise in the current study, no change in body weight or overt behaviour were observed in QD-administered animals compared to the control group.

**Fig 9:**
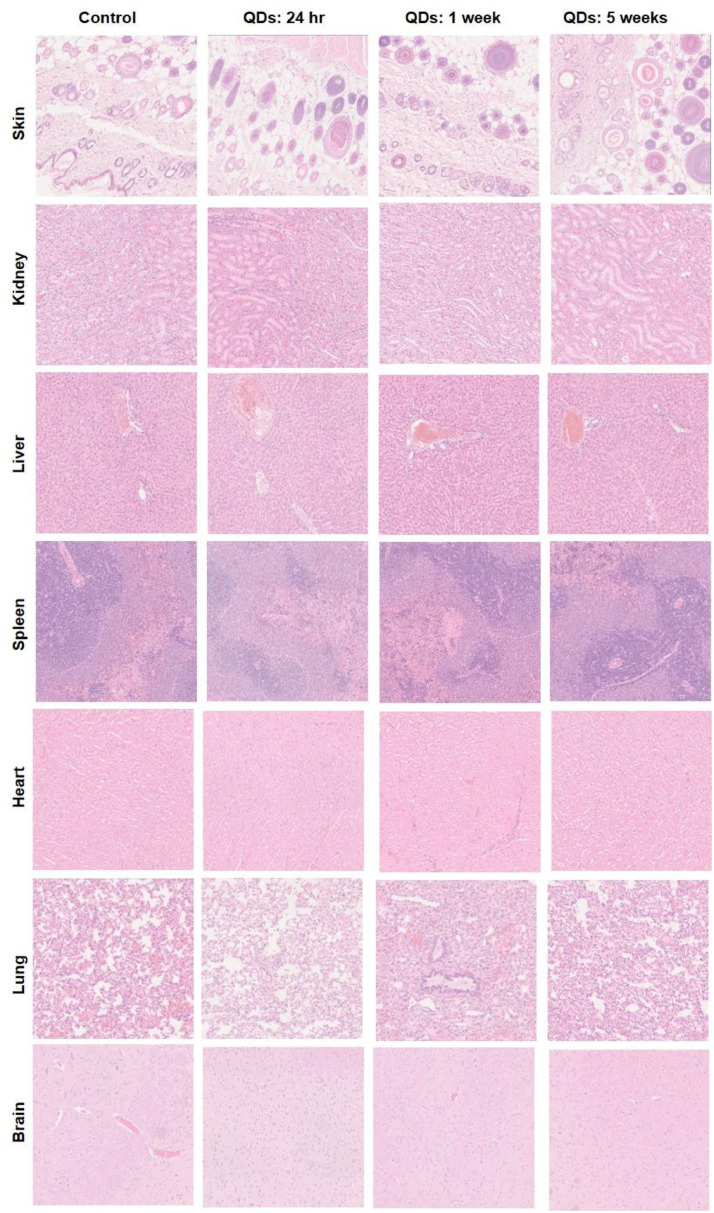
Representative H & E stained images of major organs including skin, kidney, liver, spleen, heart, lung and brain from the control (untreated) and QDs-injected rats following intravenous injection at 48 mg/kg at 24 hr, 1 week, and 5 weeks post-injection.

**Fig 10:**
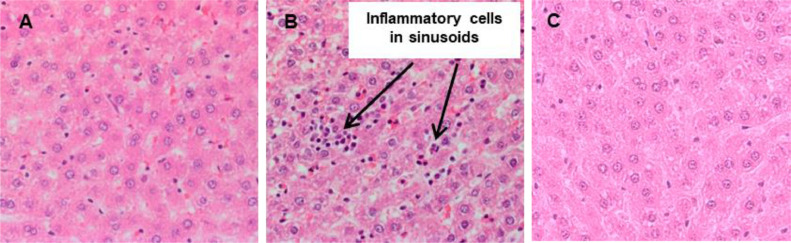
H & E stained liver sections in (A) control rats, (B) rats administered with QDs at 48 mg/kg 1 week post-injection, and (C) rats administered with QDs at 48 mg/kg 5 week post-injection.

Histopathological assessments of various tissues were employed to investigate any tissue injury induced by QDs. Representative histology images are shown in Fig. 9 for the higher QDs dose group (48 mg/kg). Several studies on cadmium-based QDs revealed that accumulation of QDs in the liver results in pathological changes and functional impairment of the liver (Liu *et al.*, 2011; Tang *et al.*, 2013; Lu *et al.*, 2016). In this study, 1 week after QDs injection at the highest dose of 48 mg/kg mild and transient accumulation of the inflammatory cells was observed in the liver (Fig. 10). These adverse findings consisted of inflammatory cell infiltration, typically multifocal in distribution, but occasionally more diffusely within the hepatic sinusoids. The infiltrates were composed predominantly of macrophage (probably activated Kupffer cells) but a few neutrophils were also present. In general, hepatocytes were not involved in the reaction but an occasional necrotic cell was observed at the margin of the infiltrates. No findings were observed in the liver at 24 h. This is presumably due to insufficient time for the inflammatory response to become evident/established. No findings were observed at 5-week time points, which indicates that full recovery has occurred. The difference in response between liver and spleen is interesting in view of the comparable uptake observed in each organ. We speculate that the spleen has a well established and active macrophage population that can process the QDs, whereas the Kupffer cells in the liver are smaller and need to undergo activation following exposure to the QDs, thereby resulting in morphological differences.

Histopathological assessment of the spleen revealed no sign of an inflammatory response or pathological changes, despite the relatively high uptake in this organ. Several studies have reported inflammation and granuloma formation in the lung following the administration of cadmium-based QDs (Ho *et al.*, 2013; Roberts *et al.*, 2013; Lee *et al.*, 2015). However, in our study no inflammation was noted in lungs. Histopathological analysis of kidney, brain, heart and skin did not show any histopathological changes or abnormalities.

No statistically significant changes were observed for any of the haematological markers in the QD-injected groups, as compared to control animals (Figure S1). The slight increase in WBC levels in QD-injected rats with 48 mg/kg at 1 week post-injection was consistent with the inflammatory cell infiltration in the liver at 1 week post-injection time. However, no statistically significant changes in WBC levels was observed in rats at 24-h and 5-week post-injection time points.

Clinical biochemistry tests were performed to evaluate organ function. Slightly higher levels of AST and ALT were observed at 24 h and 5 weeks with the high dose of QDs, as compared to the control group (Figure S2). No trend in ALP levels were observed compared to the control group. As depicted in Figure S2, the amount of total protein in QDs-treated animals showed similar trends to those from the control group. Briefly, our studies showed a slight increase in liver enzymes (ALT, AST and ALP), in association with increased WBC (48 mg/kg) at the 1-week time-point, which is consistent with a low-grade, sub-lethal disturbance of hepatocytes (Figure S1). The minor nature of this injury is confirmed by the presence of normal circulating total protein/albumin levels, which indicate no alteration in liver function and therefore the findings observed in the liver histopathology were reversible and non-adverse. These enzymatic changes are probably due to the degradation and breakdown of QDs following their accumulation in the liver. Consequently, it is likely that the small increase in WBC represents a response to the event in the liver. Increase in the number of WBC have been reported in other studies following intravenous injection of QDs, which have been attributed to the inflammatory response. There were no other haematological differences between QD-injected animals and those in the control group.

Kidney function was assessed by measuring blood urea nitrogen (BUN) and creatinine (Cr). Similar to previous findings in haematology and biochemistry, our study showed that the levels of BUN and Cr in QDs-treated animals do not differ from control groups, suggesting that there was no effect on renal function (Figure S2).

## 5 Conclusions

In conclusion, the use of a population pharmacokinetic modelling approach has enabled the characterisation of the *in vivo* fate of regulated heavy metal-free indium-based QD nanoparticles in mice. Our results also demonstrated their biocompatibility, without organ damage following systemic administration of the QDs at a relatively high dose of 48 mg/kg. These results supports their potential use for future biomedical applications. Most importantly, this study provided insight into tissue distribution and equilibration kinetics in two major organs despite the sparseness of the experimental data. In contrast to the standard descriptive summary of exposure levels across different organs over time, modelling of the experimental data in serum and organs was very informative, in that it indicates that QDs redistribute back into blood circulation at different rates prior to being eliminated slowly from the body via biliary and renal routes, as assessed by indium concentrations in faeces and urine.

Even though compartmental modelling does not fully represent pre-specified physiological processes, together with toxicology data, it offers a robust basis for extrapolation of disposition characteristics from animal to humans, supporting the dose rationale in clinical studies. Identifying the dose range to be evaluated in humans before designing a clinical protocol is a critical milestone in early phase clinical studies. Ultimately, an approach can be envisaged in which the proposed models are extended to other species as well as for comparison of circulating nanoparticles, enabling a more systematic evaluation of the *in vivo* disposition characteristics of such nanoparticles.

## Authorship contribution statement

**Elnaz Yaghini:** Conceptualization, conducting all experiments, data curation, formal analysis, writing original daft & editing, funding acquisition; **Elisa Tacconi:** Pharmacokinetic data analysis; **Andrew Pilling:** Reviewing histopathology slides, manuscript reviewing and editing; **Paula Rahman:** Synthesis of quantum dots; **Joe Broughton:** Characterisation of quantum dots; **Imad Naasani:** Provision of quantum dots, Manuscript reviewing and editing, Funding acquisition; **Mohammed R.S. Keshtgar:** Funding acquisition; **Alexander MacRobert:** Funding acquisition, manuscript reviewing and editing, **Oscar Della Pasqua:** Pharmacokinetic data analysis, reviewing and editing of manuscript.

## Declarations of interest

None of the authors have a conflict of interest regarding the present study or have anything to disclose.

## Appendix Supplementary materials

Image, application 1
