# Supplementary material for: Population pharmacokinetic modelling of indium-based quantum dot nanoparticles: preclinical in vivo studies
Source: Eur J Pharm Sci. 2021 Feb 1;157:105639. doi: 10.1016/j.ejps.2020.105639 (PMC8214104; doi:10.1016/j.ejps.2020.105639)
Supplement: Supplementary file 1 [file mmc1.pdf]

Population pharmacokinetic modelling of indium-based quantum dot nanoparticles: preclinical *in vivo* studies

Elnaz Yaghini [a]*, Elisa Tacconi [b], Andrew Pilling [c], Paula Rahman [d], Joe Broughton [d], Imad Naasani [d], Mohammed R.S. Keshtgar [a], Alexander J. MacRobert [a], Oscar Della Pasqua [b]

[a] UCL Division of Surgery and Interventional Science, University College London, Charles Bell House, 43-45 Foley Street, London, W1W 7TS, UK

[b] Clinical Pharmacology and Therapeutics Group, University College London, School of Pharmacy, BMA House, Tavistock Square, London, WC1H 9JP, UK

[c] ToxPath Consultancy Ltd, Church Road, Wingfield, Diss, IP21 5RA, UK

[d] Nanoco Technologies Ltd, 46 Grafton Street, Manchester M13 9NT, UK

*Corresponding author: Elnaz Yaghini (email: elnaz.yaghini@ucl.ac.uk)

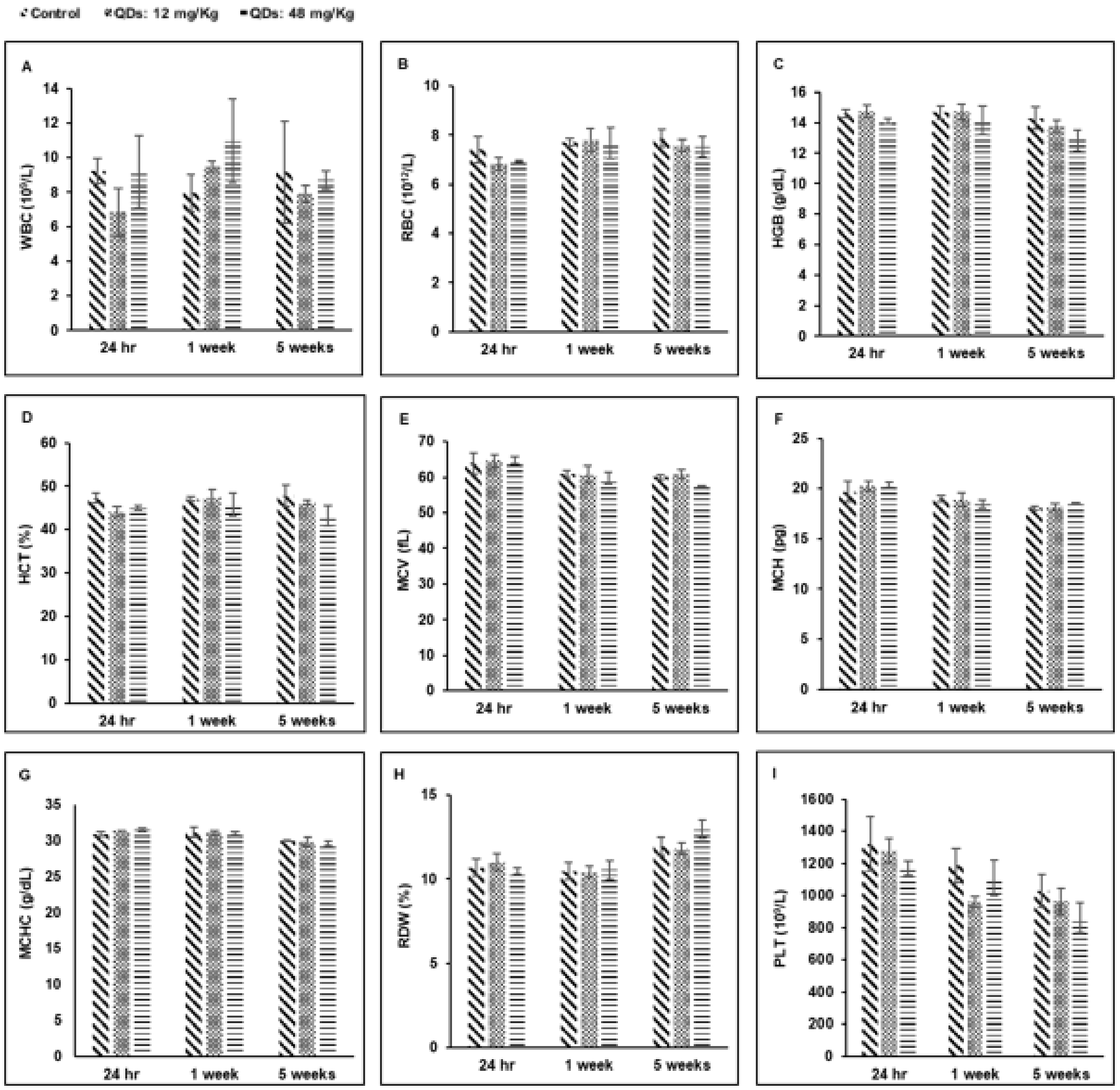

Figure S1. Haematology results of the quantum dot nanoparticles injected intravenously into the rats (n = 3). The results show mean and standard deviation of white blood cells (WBC), red blood cells (RBC), haemoglobin (HGB), haematocrit (HCT), mean corpuscular volume (MCV), mean corpuscular haemoglobin (MCH), mean corpuscular haemoglobin concentration (MCHC), red blood cell distribution width (RDW), platelet (PLT).

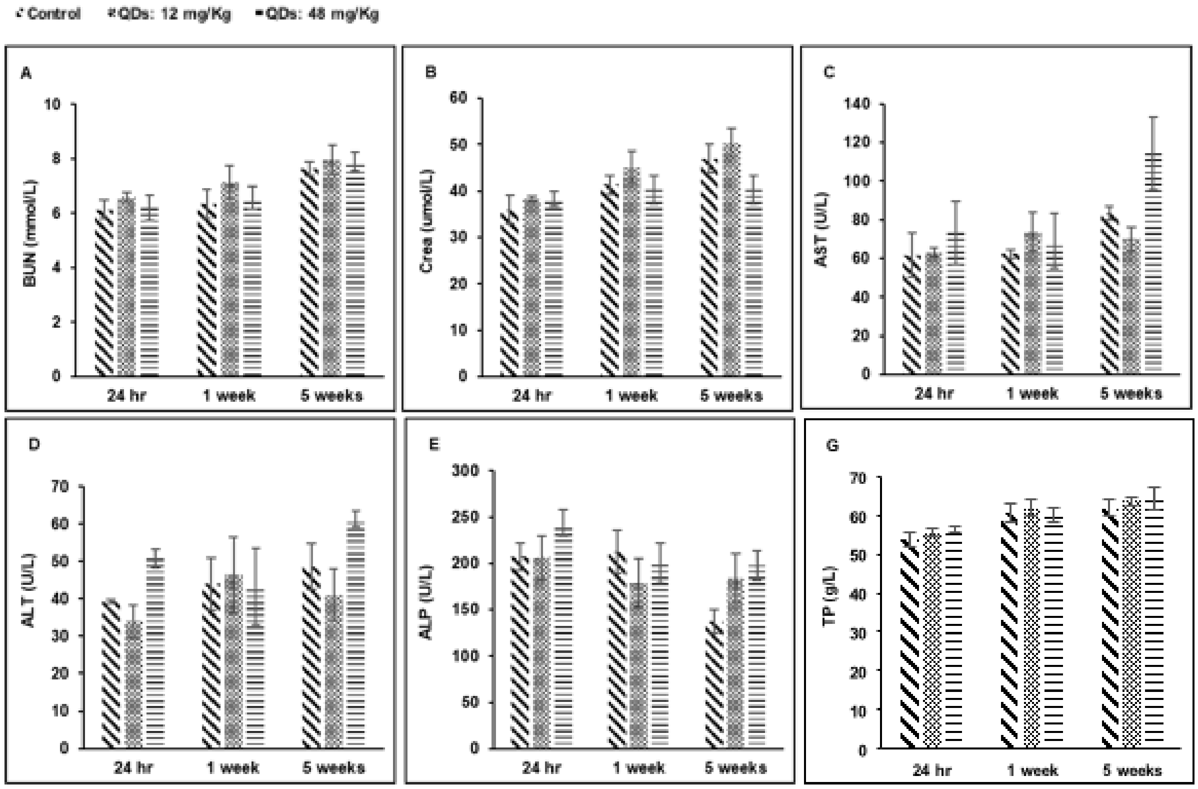

Figure S2. Blood biochemical results of the quantum dot nanoparticles following intravenous into rats (n = 3). The results show mean and standard deviation of (A) blood urea nitrogen (BUN), (B) creatinine (Crea), (C) aspartate transaminase (AST), (D) alanine transaminase (ALT), (E) alkaline phosphatase (ALP), (F) total protein.
